# Quality of Life and Migraine Disability among Female Migraine Patients in a Tertiary Hospital in Malaysia

**DOI:** 10.1155/2015/523717

**Published:** 2015-01-08

**Authors:** Munvar Miya Shaik, Norul Badriah Hassan, Huay Lin Tan, Siew Hua Gan

**Affiliations:** ^1^Human Genome Centre, School of Medical Sciences, Universiti Sains Malaysia, 16150 Kubang Kerian, Kelantan, Malaysia; ^2^Department of Pharmacology, School of Medical Sciences, Universiti Sains Malaysia, 16150 Kubang Kerian, Kelantan, Malaysia

## Abstract

*Background*. Disability caused by migraine may be one of the main causes of burden contributing to poor quality of life (QOL) among migraine patients. Thus, this study aimed to measure QOL among migraine sufferers in comparison with healthy controls. *Methods*. Female diagnosed migraine patients (*n= 100*) and healthy controls (*n=100*) completed the Malay version of the World Health Organization QOL Brief (WHOQOL-BREF) questionnaire. Only migraine patients completed the Malay version of the Migraine Disability Assessment questionnaire. *Results*. Females with migraines had significantly lower total WHOQOL-BREF scores (84.3) than did healthy controls (91.9, *P<0.001*). Similarly, physical health (23.4 versus 27.7, *P<0.001*) and psychological health scores (21.7 versus 23.2, *P< 0.001*) were significantly lower than those for healthy controls. Seventy-three percent of patients experienced severe disability, with significantly higher number of days with headaches (13.8 days/3 months, *P< 0.001*) and pain scores (7.4, *P< 0.013*). Furthermore, migraine patients with lower total QOL scores had 1.2 times higher odds of having disability than patients with higher total QOL scores. *Conclusions*. The present study showed that migraine sufferers experienced significantly lower QOL than the control group from a similar population. Disability was severe and frequent and was associated with lower QOL among the migraine patients.

## 1. Background

Migraines are prevalent globally and are one of the leading neurological reasons sought for medical care [1]. According to the World Health Organization, migraine is ranked 19th among all diseases causing disability and is the 12th leading cause of years lived with disability among females of all ages worldwide [2]. Migraine is generally considered a disabling disease that can significantly reduce the quality of life (QOL) of persons affected by it [3, 4]. It has been reported that female migraine sufferers tend to outnumber male sufferers by nearly 3 to 1 [5]. In 1996, the prevalence of migraines in Malaysia was 9% [6]. However, there is no recent study reporting the prevalence of migraine in Malaysia.

Migraine is a recurrent, severe, and throbbing headache affecting one side of the head. It is usually associated with nausea, vomiting, photophobia, or phonophobia. Most migraineurs are chronically affected by migraine and have recurrent episodic attacks, which have the potential to progress to more frequent and severe attack patterns [7]. The frequency and severity of headaches can progress over months or years to chronic migraine, in which attacks tend to occur for at least 15 days in a month [7]. These attacks are associated with substantial functional impairments [8–10], which may include both physical and psychological effects [11] and impact academic [12], occupational [13], social, and family lives [8, 10, 14]. These impairments can occur during or between the migraine attacks. Generally, migraineurs report poorer subjective well-being and reduced quality of life during their pain-free periods than do age- and sex-matched healthy controls [10, 14, 15].

In a review article, Buse et al. (2009) reported that more than half of migraine patients need bed rest during a migraine attack [9]. Three quarters of patients psychologically assumed that they would have migraines for the rest of their lives and the remaining quarter of patients worried about migraines between attacks. Migraine patients with psychological symptoms tend to take pain medications before any symptoms, which contributes to the overuse of these medications. They also reported lower health-related QOL and psychological condition with migraine [9]. Unfortunately, clinicians tend to focus on treatment frequency and severity of migraine and often fail to address the overall functional impairments associated with this disorder [11]. The majority of clinicians did not recognize the degree and scope of impairment caused by migraine [16, 17], which sometimes contributed to missed opportunities for giving effective acute management during migraine attacks and prevented effective pharmacotherapies [16, 17]. Severe migraine headaches often remained despite the availability of effective treatment and management options.

Measurement of QOL and disability has emerged as an important complementary approach that can aid in the management of migraine. Assessing a patient's QOL is an effective way of measuring the burden of migraine as it focuses on activity limitations or temporary disabilities. Many studies reported migraine disability by estimating the time lost due to migraine, including reduced effectiveness and capability of doing daily chores [18–22]. Several instruments with good reliabilities and validities have been designed to identify the comorbid psychological disturbances [23–25], measure the burden and disability [26–28], and assess the effects of migraine on QOL [9, 29].

The World Health Organization QOL Brief (WHOQOL-BREF) questionnaire is a short, simple, and fast instrument for the measurement of health-related QOL. This self-administered questionnaire is widely accepted for generic use in a variety of cultural settings (28) and is available in Malay [30]. It is a 26-item abbreviated version of the World Health Organization QOL questionnaire and is regarded to be very important for assessing QOL. The WHOQOL-BREF questionnaire has four domains (physical health, psychological health, social relationships, and environment), whose scores can depict the profile of the QOL of migraine patients [29]. There are two parts that can be separately examined: part 1 investigates an individual's overall perception of QOL and part 2 examines the individual's overall perception of health.

Migraine disability is commonly measured using the Migraine Disability Assessment (MIDAS) questionnaire [26]. This short, self-administered questionnaire has advantages over other instruments as it measures the number of days lost at work, home, and recreational activities [26, 31, 32]. There is a validated Malay version of the MIDAS questionnaire [33], and it was applied in this study.

To date, there is no published study on QOL and disability among Malaysian migraine patients. In this study, we aimed to measure QOL among migraine patients. Disability among migraine patients and the associations between migraine disability and QOL were also investigated.

## 2. Methods

**Figure 1:**
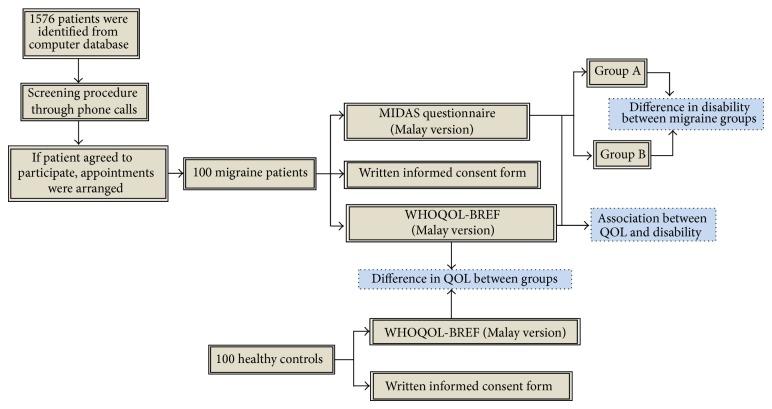
Flowchart of data collection. Group A comprised patients with either grade I or grade II migraine based on the MIDAS questionnaire who require only over-the-counter analgesics to reduce migraine disability. Group B included patients from grade III or IV who require migraine-specific treatments. MIDAS: Migraine Disability Assessment, QOL: quality of life, and WHOQOL-BREF: World Health Organization's Quality of Life Brief questionnaire.

This study was conducted in Hospital Universiti Sains Malaysia (HUSM), which is a multispecialty teaching hospital located in Kubang Kerian, Kelantan, Malaysia, between January and November 2013. Initially, 1576 migraine patients were identified from the computerized patient database over the previous three years (Figure 1). The inclusion criteria include patients between 15 and 60 years old diagnosed with migraine for more than one year. Pregnant women and migraine patients with any neurological or cardiovascular diseases or history of trauma were excluded. During this phase, individual phone calls were made to patients who suffered migraine attacks and those who were admitted to the hospital or attended the migraine clinic over the past three years. If patients agreed to participate, appointments were arranged for visits to the neurology clinic, where their migraines were confirmed as per the International Classification of Headache disorders (ICHD-II) criteria for migraine [34].

After the initial screening process against the inclusion and exclusion criteria, the patients were verbally informed about the purpose of the study and consenting patients were asked to complete the written informed consent forms. All participants were then examined by a neurologist to confirm their migraine diagnosis. Sociodemographic information was completed by the researcher, but the Malay versions of the WHOQOL-BREF and MIDAS-M questionnaires were self-administered by the patients.

### 2.1. Assessment of QOL

Total QOL of individuals with and without migraine headaches was measured using the Malay version of the WHOQOL-BREF questionnaire [30]. Individual domain scores (physical health, psychological health, social relationships, and environment) were also calculated as per instructions in the WHOQOL-BREF questionnaire [29]. The scores ranged from 24 to 120 for the total QOL and 7 to 35 for the physical health, 6 to 30 for the psychological health, 3 to 15 for the social relationships, and 8 to 40 for the environment domains. Higher scores indicate better QOL.

### 2.2. Assessment of Disability

In this study, migraine related disabilities were assessed with the Malay version of the MIDAS questionnaire [35]. This questionnaire measures the influence of headaches on three domains of activity (work, household work, and nonwork activities) over the preceding three months, with scores ranging from 0 to 92. The MIDAS score is obtained by totaling the scores of the three domains to produce a sum ranging from 0 to 276. Four disability grades are assigned based on the total scores: grade I (0–5, indicating minimal or infrequent disability), grade II (6–10, indicating mild or infrequent disability), grade III (11–20, indicating moderate disability), and grade IV (21 or more, indicating severe disability). The total MIDAS is the aggregated number of days with less than 50% predictability at work, school, and home or in recreational gatherings [26].

To assess migraine disability among migraine patients using MIDAS questionnaire, migraine patients were divided into two groups (groups A and B) based on the criteria as recommended by Stewart et al. [26]. Group A comprised patients having either grade I or grade II migraine based on the classification as determined from the MIDAS questionnaire requiring only over-the-counter (OTC) analgesics to reduce their migraine disabilities. Group B comprised patients from grade III or IV who require migraine-specific treatments. However, we have not collected the medication information prescribed to the migraine patients.

## 3. Sample Size

The sample size was calculated based on the prevalence of headache [6] using the two-proportion formula for the comparison of QOL between migraine patients and healthy subjects with the power of 0.80 and alpha of 0.05. In this study, the sample size was determined using PS-Power and Sample Size Calculation, version 3.0.43 (Vanderbilt University, Tennessee, USA) [36], and was found to be 94 subjects for each case and control group after including 20% dropouts.

### 3.1. Data Collection

A total of 100 female migraine cases and 100 nonmigraine female volunteers completed the WHOQOL-BREF questionnaire and only the migraine patients (Figure 1) completed the Malay version of the Migraine Disability Assessment (MIDAS) questionnaire. Because the pharmacotherapy for migraine largely depends on migraine severity, the patients were divided into two groups: group A was comprised of patients with either grade I or grade II migraine based on the MIDAS questionnaire who required only over-the-counter analgesics to reduce migraine disability and group B included patients of migraine grade III or IV who required migraine-specific treatments. The study was approved by the Universiti Sains Malaysia Research and Ethical committee (ethical number: USMKK/PPP/JEPeM 231.3.(08)) and complies with the Declaration of Helsinki.

### 3.2. Statistical Analysis

Independent *t*-tests were used to compare the sociodemographics, overall perception of QOL, and health between the migraine patients and the nonmigraine controls. For the comparison of the total QOL and individual domain scores using the WHOQOL-BREF questionnaire among the migraine patients and the nonmigraine controls, analysis of covariance (ANCOVA) was used. For the measurement of disability using MIDAS-M questionnaire among groups A and B of migraine patients, independent *t*-tests were used. Finally, multiple logistic regression was used to determine the associations of migraine disability with QOL among the migraine patients. Analysis was performed using IBM SPSS version 20.0 software (IBM Corporation, New York, USA).

## 4. Results

### 4.1. Sociodemographics

All the subjects successfully completed the WHOQOL-BREF and MIDAS questionnaires. None had any difficulties in understanding or answering any parts of the questionnaires. No significant difference was found between the migraine patients and the healthy control groups except for duration of education. The mean duration of education among the migraine patients was significantly lower (13.5 versus 14.9 years, *P* < 0.001) than the healthy controls. Only 28 patients (having ≥15 days headache for >3 months) were recognized as chronic migraineurs.

### 4.2. Measurement of QOL Using WHOQOL-BREF Questionnaire

The overall perception score of QOL among the migraine patients was significantly lower (3.5 versus 3.9, *P* < 0.001) than the nonmigraine healthy controls. Similarly, the overall perception of health among the migraine patients was significantly lower (3.1 versus 3.8, *P* < 0.001) than the nonmigraine healthy controls (Table 1).

The mean total QOL scores were significantly lower among the migraine patients than the healthy controls before (83.4 versus 91.9, *P* < 0.001) and after (84.3 versus 91.9, *P* < 0.001) adjusting for age and duration of education. Similarly, following adjustments for age and duration of education, the female migraine patients still had significantly lower physical health (23.4 versus 27.7, *P* < 0.001) and psychological health scores (21.7 versus 23.2, *P* < 0.001) than the healthy controls. The social relationships and environmental domain scores were lower among the female migraine patients than the healthy controls, but these differences were not statistically significant (Table 2).

### 4.3. Measurement of Disability Using the MIDAS Questionnaire

Using the MIDAS questionnaire, a total of 27% and 73% of migraine patients were classified into group A (grade 1 or 2) and group B (grade 3 or 4), respectively (Table 3). The mean age of patients in group A was 28.2 years (11.4), and the mean age in group B was 27.9 years (9.1). There was no significant difference between the groups.

For the mean migraine disability score, group B patients with moderate to severe disability scored significantly higher (33.7 versus 5.2, *P* < 0.001) than group A patients with moderate to severe disability. The migraine patients from group B reported more days with headache (13.8 days) than those from group A (3.6, *P* < 0.001). The pain scores were also significantly higher among those from group B (7.4) than those from group A (6.4, *P* < 0.013).

### 4.4. The Association between QOL and Migraine Disability

There were no significant differences in the total QOL or psychological health, social relationships, or environment domain scores between group A and B migraine patients based on the WHOQOL-BREF questionnaire (Table 4). However, the physical health domain score among group A patients was significantly higher (24.8 versus 22.7, *P* = 0.009) compared with those of group B.

### 4.5. The Association of Disability and QOL with Migraine

Migraine patients with higher MIDAS scores have 2.7 times higher odds of having disability (CI = 1.43–5.01, *P* = 0.002) than do patients with lower MIDAS scores. Migraine patients with lower total QOL scores have 1.2 times higher odds of having disability (CI = 1.001–1.45, *P* = 0.049) than do patients with higher total QOL scores (Table 5). Multiple logistic regression indicated that the MIDAS score and total QOL were significantly correlated for migraine patients without any interaction or multicollinearity problems.

## 5. Discussion

Measurements of QOL and disabilities have emerged as important complementary approaches for the evaluation of the burden of headaches [25, 31, 37, 38]. This is the first study in Malaysia to compare QOL and disability between migraine sufferers and nonmigraine controls. The Malay versions of the WHOQOL-BREF and MIDAS questionnaires were easy to administer and can be completed quickly. No subjects had any difficulty using the instruments, indicating a high quality of the questionnaires.

In this study, the overall perception score of QOL and health was significantly lower among migraine patients. This finding is consistent with those from other studies [39–41] that reported a lower perception of QOL and health. The migraine sufferers had substantial and statistically significantly lower total QOL scores and physical health and psychological health domain scores than the healthy control group. In another study conducted among migraine patients in the USA [25], the total QOL, physical health, and social functioning scores of the migraine patients were substantially lower than the published norms. A similar study conducted among the Dutch population [42] reported diminished functioning and well-being among migraineurs. Consistently, total QOL, physical health, and psychological health scores were significantly lower among UK [43], French [20], Italian [44], and Indian [45] migraine patients than nonmigraine controls.

Lower QOL could be attributed to underdiagnosis or underestimation of migraine, lack of awareness of migraine triggers, and poor management of migraine headaches. After migraine attacks, patients tend to be physically weak, which may disrupt their daily routines. Moreover, it has been reported that the majority of migraine patients tend to receive treatment from general practitioners rather than from migraine specialists [46]. Furthermore, the majority of clinicians or general practitioners often underestimate the burdens caused by migraine, which in turn may also affect migraine management [16, 17].

Most migraine patients (73%) in the present study had severe migraine disability (grades III and IV). The group with severe disability reported significantly higher MIDAS scores, which signified a high number of days with less than 50% of predictability at work, school, and home or in recreational gatherings. The total number of days with headache was significantly higher among the severe migraine disability group than the moderate disability group. This result is consistent with the studies conducted in Taiwan [47], USA [48], Korea [49], and Italy [50]. Moreover, the pain score was also significantly higher among the severe migraine disability group. A higher number of days with headaches with severe pain intensity were significantly associated with higher disability among Taiwanese [47], USA [48], Korean [49], and Italian [50] migraine populations. The severity and extent of headaches due to migraine can be major determinants of the burden of migraine. Severe migraine disability patients require migraine-specific treatments as they have significantly more days with headache pain than the moderate disability group. Possible contributing factors could be underdiagnosis in the early years of migraine.

In this study, a higher pain threshold in combination with a higher number of days with headache among migraine patients with severe disability was significantly associated with a lower physical health score. Therefore, disability due to severe headache pain and frequency of pain may lead to physical weakness, which may pose a hurdle to normal physical activities at home and at the workplace. However, other health score domains were not significantly different between the groups, perhaps due to similar psychological, social, and environmental conditions.

Severe disability and higher MIDAS scores were significantly associated with lower total QOL scores. Similarly, a study among migraine populations in Taiwan reported significantly lower total QOL scores and higher MIDAS scores [47] among patients with severe disability. Consistently, the International Burden of Migraine Study also reports significantly lower total QOL scores and higher MIDAS scores among migraine patients due to greater health care resource utilization among migraine patients [48]. In another study from England, moderate and severe disability groups reported a greater reduction in total QOL than the group with mild disability [43]. Therefore, assessing disability and QOL is very important to help clinicians in making more appropriate prescribing choices for migraine pharmacotherapy.

*Limitations of the Study.* The study sample was limited to female migraineurs because no male patients registered at the clinic during the study period. The higher number of female migraineurs seen was consistent with the report by Lipton et al. (2003) [51], which reported a higher prevalence of female than male migraine sufferers (three times more) [5]. Several studies reported that gender differences have an impact on QOL [11] and the frequency [52], disability [53], and treatment [54] of migraines. Therefore, the findings may not be applicable to male migraine patients.

The sample was restricted to Malay patients because the majority of Kelantanese people are Malay. Other races, such as Chinese and Indian, constitute a lower proportion of the population in Kelantan. Migraine is a genetically associated disease [55] and there is a possibility that genetic differences may modify outcomes among migraine patients.

Duration of education was significantly lower among migraine patients than healthy controls. This may indicate lower self-awareness among migraine patients. A study conducted in the USA [56] reported that higher education among headache patients creates some awareness of the possible types of migraine triggers and contributes to proper management of headache. High QOL scores were reported among patients who were cautious about the management of their headaches. In this study, we did not collect the medication information prescribed to the migraine patients. Nevertheless, we have utilized multivariate analysis to adjust for duration of education and other variables to minimize this confounding factor. Increased age can also cause more disability among migraine patients due to decrease in physical strength. However, there was no significant difference in terms of age between migraine patients and healthy controls in this study. Further studies are recommended to determine other factors associated with migraine disability.

## 6. Conclusion

The present study indicates that migraine sufferers have substantial and statistically significant reductions in physical and psychological QOL in comparison with a contemporaneous control group drawn from a similar population. Disability was severe among the migraine patients and was associated with lower QOL. Therefore, healthcare professionals should routinely evaluate QOL and related disability to determine whether patients are receiving effective treatment and whether any additional treatment strategies are warranted to improve QOL.

*Clinical Implications.* Consider the following:

- measurements of QOL and migraine disability being performed for the first time in Malaysia,
- comparison of total QOL and other individual domains like physical health and psychological health between migraine patients and healthy subjects,
- association of QOL and migraine disability,
- association of disability and QOL with migraine.

## Figures and Tables

**Table 1:** Perception of QOL and health among Malay migraine patients using WHOQOL-BREF questionnaire.

	Mean (SD)		*P* value
	Nonmigraine (*n* = 100)	Migraine (*n* = 100)	
Overall perception of quality of life	3.9 (0.6)	3.5 (0.7)	**0.01**
Overall perception of health	3.8 (0.6)	3.1 (0.7)	**<0.001**

SD: standard deviation.

**Table 2:** Comparison of total QOL scores between migraine and nonmigraine subjects.

	Mean (SD)		Mean differences	*P* value
	Nonmigraine	Migraine		
	(*n* = 100)	(*n* = 100)		
TQOL score				
Nonadjusted	91.9 (8.8)	83.4 (11.4)	8.5 (5.6, 11.3)	<0.001
Adjusted	91.9 (89.8, 93.9)	84.3 (82.2, 86.4)	7.6 (4.6, 10.5)^§^	<0.001
Physical health score				
Nonadjusted	27.7 (3.1)	23.3 (3.5)	4.4 (3.5, 5.3)	<0.001
Adjusted	27.7 (27.0, 28.4)	23.4 (22.7, 24.1)	4.3 (3.3, 5.3)^§^	<0.001
Psychological health score				
Nonadjusted	23.3 (2.4)	21.5 (3.2)	1.8 (1.12, 2.56)	<0.001
Adjusted	23.2 (23.6, 23.8)	21.7 (21.1, 22.3)	1.51 (1.03, 2.3)^§^	<0.001
Social relationships score				
Nonadjusted	11.3 (1.6)	10.8 (1.6)	0.5 (0.02, 0.9)	0.037
Adjusted	11.3 (11.0, 11.6)	10.9 (10.6, 11.2)	0.4 (−0.7, 0.9)^§^	0.094
Environment score				
Nonadjusted	29.7 (3.7)	28.9 (3.3)	0.8 (−0.2, 1.75)	0.13
Adjusted	29.0 (28.3, 30.3)	29.0 (28.3, 29.8)	0.5 (−0.5, 1.57)^§^	0.31

SD = standard deviation.

Non-adjusted: Independent *t*-test applied.

Adjusted: ANCOVA applied (adjusted for age and duration of education).

^§^Adjusted means difference (95% confidence interval), Bonferroni adjustment applied.

**Table 3:** Disability among female migraine patients.

Variable	Mean (SD)		*t* statistic (df)	*P* value^*^
	Group A (*n* = 27)	Group B (*n* = 73)		
Age (years)	28.2 (11.4)	27.9 (9.1)	0.15 (98)	0.884
Duration of education (years)	13.2 (2.4)	13.6 (2.8)	−0.70 (98)	0.485
Migraine Disability Assessment Score	5.2 (3.5)	33.7 (25.9)	−5.68 (98)	**<0.001**
Total number of days with headache	3.6 (2.7)	13.8 (10.7)	−4.83 (98)	**<0.001**
Pain scale score	6.4 (2.2)	7.4 (1.6)	−2.53 (98)	**0.013**

Notes: ^*^independent *t*-test.

Group A comprised patients with either grade I or grade II migraine based on MIDAS questionnaire who require only over-the-counter analgesics to reduce migraine disability.

Group B included patients from grade III or IV who required migraine-specific treatments.

**Table 4:** Association of QOL with migraine disability.

Variable	Mean (SD)		*t* statistic (df)	*P* value^*^
	Group A (*n* = 27)	Group B (*n* = 73)		
Physical health score	24.8 (3.3)	22.7 (3.5)	2.655 (98)	**0.009**
Psychological health score	22.0 (3.2)	21.3 (3.2)	0.947 (98)	0.346
Social relationships score	10.9 (1.6)	10.8 (1.5)	0.411 (98)	0.682
Environment score	29.0 (3.7)	28.9 (3.2)	0.251 (98)	0.803

Total QOL scores	86.7 (9.9)	82.2 (11.8)	1.781	**0.078**

Notes: ^*^independent *t*-test.

Group A comprised patients with either grade I or grade II migraine based on MIDAS questionnaire who require only over-the-counter analgesics to reduce migraine disability.

Group B included patients from grade III or IV who required migraine-specific treatments.

**Table 5:** Association of disability and QOL with migraine.

Variables	Crude OR^*^	Adjusted OR^#^	Wald statistics^#^	*P* value^#^
	(95% CI)	(95% CI)	(df)	
MIDAS score	1.92 (1.36, 2.73)	2.68 (1.43, 5.01)	9.53 (1)	**0.002**
Total QOL	0.96 (0.93, 1.00)	1.20 (1.001, 1.45)	3.87 (1)	**0.049**

^*^Simple logistic regression and ^#^multiple logistic regression (method = Backward LR).

The model reasonably fits well. Model assumptions are met. There are no interaction or multicollinearity problems.

## Abbreviations

MIDAS: Migraine Disability Assessment
MIDAS-M: Migraine Disability Assessment Malay version
QOL: Quality of life
WHO: World Health Organization
WHOQOL-BREF: WHO Quality of life Brief questionnaire.
